# TAo TAVI + OPCABG: a new hybrid option

**DOI:** 10.1186/1749-8090-10-S1-A24

**Published:** 2015-12-16

**Authors:** Andrew Brazier, Imthiaz Manoly, Doug Fraser, Ragheb Hasan

**Affiliations:** 1 Department of Cardiothoracic surgery, Manchester Royal Infirmary, Manchester, M13 9WL, UK; 2 Department of Cardiology, Manchester Royal Infirmary, Manchester, M13 9WL, UK

## Background/Introduction

Hybrid surgical approaches are gaining popularity. Minimally invasive aortic valve replacement and percutaneous coronary intervention [PCI] is well documented. We wanted to explore the feasibility of trans-catheter aortic valve implantation [TAVI] and off pump coronary artery bypass grafts [OPCAB].

## Aims/Objectives

We proposed that in TAVI patients where PCI is not possible or has failed, trans-aortic TAVI with concomitant OPCAB would be a suitable alternative.

## Method

A 77 year old man admitted following an out of hospital cardiac arrest was found to have an occluded left anterior descending (LAD) artery, a patent stent in a dominant circumflex and a small diseased right coronary. Past medical history included myocardial infarctions on four occasions, PCI, and severe left ventricular failure. Echocardiography demonstrated severe, calcific aortic stenosis and poor left ventricular function. The multi-disciplinary team recommended TAVI. Work up identified abdominal aortic aneurysm which precluded trans-femoral TAVI approach. A perfusion scan confirmed viable myocardium in the LAD territory. He was put forward for trans-aortic TAVI with OPCAB surgery (left internal mammary artery to LAD).

## Results

The procedure was performed as planned without any complications. Post-operative echocardiogram confirmed a well-placed valve. The patient was treated for a chest infection and discharged home on 8th post-operative day. He was seen at the outpatient clinic at 8 weeks and was asymptomatic and with good exercise tolerance

## Discussion/Conclusion

Trans-aortic TAVI and OPCAB is a potential alternative to conventional aortic valve replacement and coronary artery bypass surgery as well as the hybrid approach of minimally invasive aortic valve replacement and PCI.

## Consent

Written informed consent was obtained from the patient's next of kin for publication of this abstract and any accompanying images. A copy of the written consent is available for review by the Editor of this journal.

